# Hyperbaric Hyperoxia Accelerates Fracture Healing in Mice

**DOI:** 10.1371/journal.pone.0072603

**Published:** 2013-08-14

**Authors:** Shigeo Kawada, Eiji Wada, Ryoichi Matsuda, Naokata Ishii

**Affiliations:** 1 Department of Life Sciences, Graduate School of Arts and Sciences, The University of Tokyo, Tokyo, Japan; 2 Future Institute for Sport Sciences, Waseda University, Tokyo, Japan; Delft University of Technology (TUDelft), Netherlands

## Abstract

Increased oxygen tension influences bone metabolism. This study comprised two main experiments: one aimed to determine the bone mineral apposition and bone formation rates in vivo under hyperbaric hyperoxia (HBO), and the other aimed to evaluate the effects of exposure to HBO on fracture healing. In experiment 1, male mice were exposed to HBO [90 min/day at 90% O_2_ at 2 atmospheres absolute (ATA) for 5 days]. In experiment 2, an open femur fracture model was created in mice, followed by exposure to HBO 5 times/week (90 min/day at 90% O_2_ at 2 ATA) for 6 weeks after surgery. In experiment 1, HBO treatment significantly increased the mineral apposition and bone formation rates in the lumbar vertebra and femur and type 1 collagen alpha 1 and alkaline phosphatase mRNA expression in the lumbar vertebra. In experiment 2, at 2 weeks after fracture, the fracture callus was significantly larger in the HBO group than in the non-HBO group. Furthermore, at 4 and 6 weeks after fracture, radiographic findings showed accelerated fracture healing in the HBO group. At 6 weeks after fracture, femur stiffness and maximum load were significantly higher in the HBO group than in the non-HBO group. Urinary 8-hydroxy-2′-deoxyguanosine and plasma calcium concentrations were not significantly different between groups. These results suggest that exposure to HBO enhances bone anabolism and accelerates fracture healing without causing oxidative DNA damage or disruption of plasma calcium homeostasis.

## Introduction

Fractures impair patients’ quality of life, and early healing of fractures is desirable. Bone regeneration after fracture occurs in the following consecutive phases: callus formation around the fracture site, osteoid formation, mineralization within the callus, and callus resorption [1], [2]. Although the molecular mechanisms underlying these phases are not fully understood, impairment of callus formation is known to delay fracture healing [2]. Mineralization of the osteoid, an essential component of the fracture healing process is regulated by alkaline phosphatase (ALP) [3]. ALP supports bone mineralization by cleaving pyrophosphate, which inhibits calcification, and by increasing the concentration of inorganic phosphates, which promotes mineralization [3].

Oxygen tension influences ALP activity; increased oxygen tension enhances ALP activity, which in turn increases bone formation and calcium deposition in vitro [4], [5]. By contrast, hypoxia suppresses both ALP activity and ALP mRNA expression in vitro [6]. Chronic hypoxic conditions delay fracture healing [7]. Moreover, hypoxia, which can result from various pathophysiological conditions such as ischemia and vascular disease, induces bone loss [8]–[10]. Yang et al. reported that hypoxia inhibited osteogenesis in human mesenchymal stem cells [11]. Therefore, these results imply that oxygen tension affects fracture healing. Barthe et al. reported that hyperbaric hyperoxia (HBO) [100% O_2_ at 2 atmospheres absolute (ATA)] for 90 min/day accelerated the repair of metaphyseal defects in the cortices of rat femurs [12]. Wray et al. reported that HBO [100% O_2_ at 2 ATA] for 6 h/day enhanced callus formation after 1-week treatment in rats [13]. Although these studies showed that HBO accelerates the histological processes underlying bone repair, it remains unclear whether restoration of biomechanical bone strength is accelerated. On the basis of previous reports, we hypothesized that HBO 1) increases the bone mineral apposition rate (MAR), the bone formation rate (BFR), and ALP mRNA expression in vivo and 2) accelerates histological and biomechanical fracture healing. In this study, we examined the effects of HBO on bone formation and fracture healing in mice.

## Materials and Methods

### Animals

Male mice (c57BL/6j) were housed in an animal room with regulated temperature (22°C), humidity (60%), and illumination cycles (12-h light and 12-h dark). They were allowed to eat commercial mouse chow (CLEA, Tokyo, Japan) and drink water ad libitum. Food consumption was measured daily. All experimental procedures were conducted in accordance with the Guide for the Care and Use of Laboratory Animals of the University of Tokyo. The study was approved by the Ethical Committee for Animal Experiments, The University of Tokyo.

### Experiment 1

**Figure 1:**
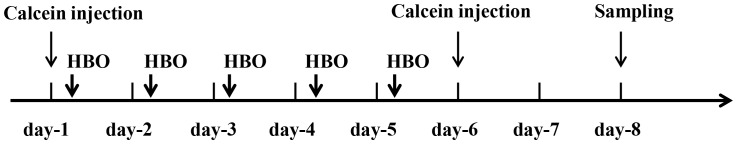
Study design in experiment 1. Calcein injection, HBO exposure, and bone sampling are indicated by arrows. A detailed description of the study design is provided in the text.

To investigate the effects of HBO on MAR and BRF in vivo, we assigned 16 mice (aged 5 weeks) to a control group (n = 8) or an HBO group (n = 8). The HBO group was exposed to HBO in a hyperbaric hyperoxic chamber (Masahide Co., Ltd., Gunma, Japan) once daily for 5 days. The mouse cages were placed in the chamber, which was automatically maintained at 90% oxygen at 2 ATA for 90 min, involving 10-min compression and 10-min decompression phases. Oxygen concentration and ATA were monitored during the experiment. To evaluate bone mineral apposition rate, the mice in both groups were double-labeled with subcutaneous injections of 10 mg/kg calcein (Sigma, St. Louis, MO) 7 and 2 days before being sacrificed. In the HBO group, exposure to HBO started immediately after the first calcein injection (Figure 1). At the end of the experimental period, the mice were killed under anesthesia to collect whole blood. The femurs and the first lumbar vertebra were removed from each mouse and fixed with 70% ethanol. The bone specimens were embedded in hydroxyethyl-methacrylate (Okenshoji Co., Ltd., Tokyo, Japan). The secondary spongiosa region of the distal femur and the lumbar vertebra were serially cross-sectioned at 10-µm thickness on a microtome. The MAR and BRF were measured using a fluorescent microscope. Each section was examined under 200× magnification, and the distance between the two calcein-labeled lines was measured at >10 randomly selected points to determine the MAR for individual cross-sections. The BRF was calculated as the product of mineralizing surface/bone surface (BS) and MAR and was expressed per BS (BRF/BS, mm^3^/mm^2^/year) [14].

### Evaluation of osteoblasts and osteoclasts

The sections were stained for ALP and tartrate-resistant acid phosphatase (TRAP) activities using a commercially available kit (TRAP/ALP stain kit; Wako Pure Chemical Industries Ltd., Osaka, Japan) according to the manufacturer’s instructions. ALP-positive nucleated cells attached to the bone were scored as osteoblasts, and TRAP-positive multinucleated cells attached to the bone were scored as osteoclasts. Five different fields of each section were examined under 400× magnification, and the percentage of bone surface covered by osteoblasts and by osteoclasts in the lumbar vertebra, i.e., the osteoblast surface per bone surface (Ob.S/BS) and osteoclast surface per bone surface (Oc.S/BS) values was determined as defined by Parfitt et al. [14].

### Quantitative real-time polymerase chain reaction

To investigate the effects of HBO on the expression of insulin-like growth factor (IGF)-1, ALP, and type 1 collagen alpha 1 (Col1A1) mRNA, we divided another 16 mice (aged 5 weeks) into a control group (n = 8) and an HBO group (n = 8). Although HBO treatments were almost the same as described above, the mice were killed under anesthesia on seventh experimental day to collect whole blood. The first lumbar vertebra was harvested, and all adherent soft tissues were carefully resected. Total RNA was isolated from the first lumbar vertebra using Isogen (Nippon Gene, Tokyo, Japan) and digested with DNase 1 (Promega, Madison, WI). The RNA was reverse-transcribed using the PrimeScript RT reagent kit (Takara, Tokyo, Japan), according to the manufacturer’s instructions. Complementary DNAs (cDNAs) for IGF-1, ALP, Col1A1, and glyceraldehydes-3-phosphate dehydrogenase (GAPDH) were amplified using SYBR Premix Ex Taq II (Takara). Following oligonucleotide primers were used to amplify each cDNA: IGF-1, forward 5′-TCACTGCCCAATTGAAATACGA-3′ and reverse 5′-TTAGGCCCAGACAGTTTAAACAAAG-3′; ALP, forward 5′-GCAGTATGAATTGAATCGGAACAAC-3′ and reverse 5′-ATGGCCTGGTCCATCTCCAC-3′; Col1A1, forward 5′-GACATGTTCAGCTTTGTGGACCTC-3′ and reverse 5′-GGGACCCTTAGGCCATTGTGTA-3′; GAPDH, forward 5′-TGTGTCCGTCGTGGATCTGA-3′ and reverse 5′-TTHCTGTTGAAGTCGCAGGAG-3′. A melt curve analysis was performed to ensure that a single polymerase chain reaction (PCR) product was amplified. GAPDH was used as an internal control for detecting changes in mRNA expression because its expression levels were almost the same between the groups. Each PCR was run using the Thermal Cycler Dice real-time system (Takara). All experiments were performed in duplicate. Data quantification was performed with the Thermal Cycler Dice real-time system software using the 2^−ΔΔ^ method [15]. Primer amplification efficacies were determined to be approximately equal.

### Experiment 2

**Figure 2:**
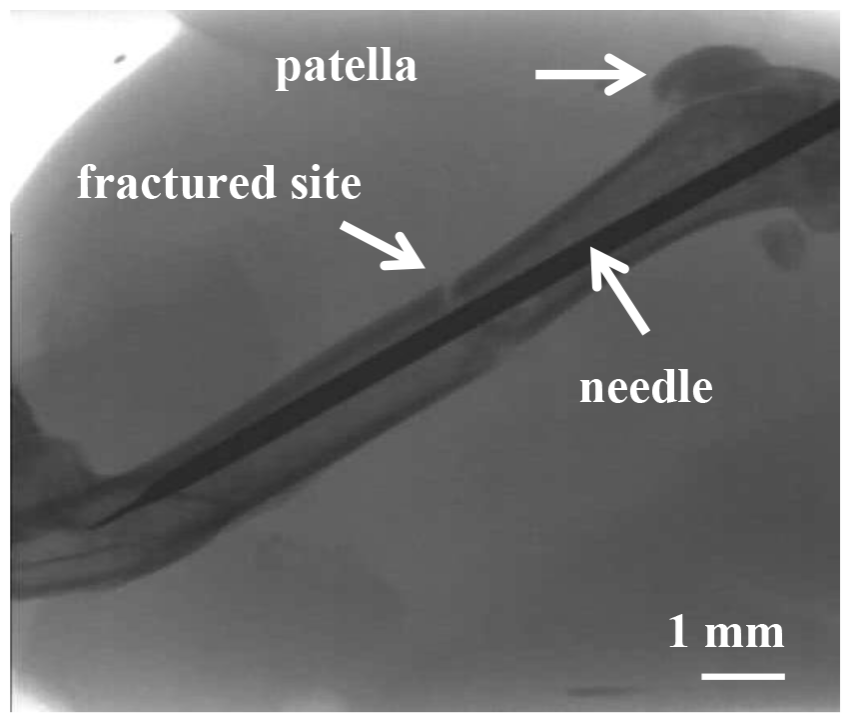
X-ray image of a fractured murine femur after stabilization with a locking needle.

To evaluate the effects of HBO on fracture healing, we developed an open femur fracture model in mice. Mice were anesthetized by inhalation of isoflurane. After scrubbing the right thigh, an 8-mm incision was made on the outside of the right thigh along the femur from the knee. The compartment between the quadriceps femoris and hamstring muscles was cut open using a surgical knife to visually confirm the femur. Then the mid portion of the femur was cut using a surgical scissors. After the cutting, the patella was dislocated to expose the femoral condyles. The intramedullary canal at the intercondylar notch was opened with a 0.5-mm-diameter trephine. A tip of 0.75-inch, 27-gauge needle was inserted directly into the medullary canal, up to the proximal end of the femur to fix the fracture, and the incision was sutured. No analgesics were administered. A representative X-ray image of the fracture model used in this study is shown in Figure 2. The same surgery was performed in the sham-operated group as a control, with the exception that the femur was not cut.

### HBO treatment for fracture healing

Seventy-two mice (aged 8 weeks) were divided as follows: 1) the sham-operated group (n = 24), 2) fracture without HBO treatment (non-HBO) group (n = 24), and 3) fracture with HBO treatment (HBO) group (n = 24). The mice in the HBO group were exposed to HBO as described in the previous experiment. The HBO treatments were commenced immediately after the surgical operation and conducted on 5 consecutive days per week. At 2 (n = 8 for each group), 4 (n = 4 for each group), and 6 (n = 12 for each group) weeks after fracture (or sham operation), the mice in each group were killed under anesthesia to collect whole blood, respectively.

### Radiography

At 2, 4, and 6 weeks after fracture, the right and left thighs of the mice in each group were harvested. Radiographs of the right and left femurs were taken at 2, 4, and 6 weeks after fracture by using an X-ray unit (SkyScan 1074, Toyo Co., Tokyo, Japan). The callus area at the fracture site was measured at 2 weeks after fracture because this area is known to peak at this time point in mice [1], [2]. The degree of callus formation was calculated as the ratio of the callus area to the femoral bone diameter at the fracture gap [16].

### Histomorphometric analyses of fracture healing

After X-ray images obtained at 2 weeks after fracture, the inserted needle was removed and all adherent soft tissues were resected. The fractured femurs were then embedded in hydroxyethyl-methacrylate (Okenshoji Co., Ltd.). Sagittal plane sections (10-µm thick) of the femur were cut using a microtome and stained with Van Gieson’s stain and ALP to assess osteoid formation (percentage area of osteoid/callus) and Ob.S/BS in the callus.

### Biomechanical testing

At 6 weeks after fracture, 6 mice from each group were used for biomechanical testing. The testing involved three-point bending tests and a testing machine (MZ-500S; Maruto Co., Tokyo, Japan) and were performed on both the fractured and contralateral nonfractured femurs. After X-ray images were obtained, the inserted needle was removed and all adherent soft tissues were resected from the fractured femur. The harvested samples were placed in a constant orientation (lateral bending) during the testing. The outer loading points of the three-point bending fixture were placed 6 mm apart, and the middle anvil was centered on the fractured point. The specimen was then bent to failure at 2 mm/min. Analog data from the load cell were outputted directly to an X-Y recorder. Structural properties were determined using load-elongation curves. The outcome measures included maximum load and stiffness, which were calculated using least-squares linear regression. Data were expressed as absolute values.

### Urine and blood analyses

To evaluate whole-body oxidative DNA damage, we measured the urinary 8-hydroxy-2′-deoxyguanosine (8-OHdG) level. 8-OHdG involves hydroxylation of guanine at the C-8 position and is one of the most abundant oxidative DNA lesions. We collected 24 h urine samples at 2, 4, and 6 weeks by placing the mice in special cages (Metabolism cage; Tecniplast, Tokyo, Japan). The collection of urine samples started 24 h after the last exposure to HBO at 2, 4, and 6 weeks of the experimental period. The urinary 8-OHdG level was determined at 6 weeks using a commercially available kit (New 8-OHdG Check; Japan Institute for the Control of Aging, Shizuoka, Japan) according to the manufacturer’s instructions. To evaluate bone resorption, we measured the urinary deoxypyridinoline (DPD) level at 2, 4, and 6 weeks after fracture using a commercially available kit (Metra DPD EIA kit; Quidel Co., San Diego, CA) according to the manufacturer’s instructions. Whole-body calcium homeostasis was assessed using plasma calcium concentration measurements at 2, 4, and 6 weeks after fracture using a commercially available kit (QuantiChrom Calcium Assay kit; BioAssay Systems, Hayward, CA) according to the manufacturer’s instructions. All samples were measured in duplicate.

### Statistical analyses

All data were expressed as mean ± SD. The differences between the variables in experiment 1 were examined by the unpaired Student’s *t*-test. The callus area, osteoid formation, femoral biomechanical testing, and 8-OHdG data in experiment 2 were analyzed by one-way analysis of variance (ANOVA) followed by the Fisher’s protected least significant difference post hoc test. The other data in experiment 2 were examined using 3 (condition) × 3 (time of measurement) ANOVA for interaction and main effects. When a statistical significance was obtained, the Fisher’s protected least significant difference post hoc test was used to identify the significant differences. An alpha of *P* < 0.05 was considered statistically significant for all comparisons.

## Results

### Effects of HBO on MAR and BRF

**Figure 3:**
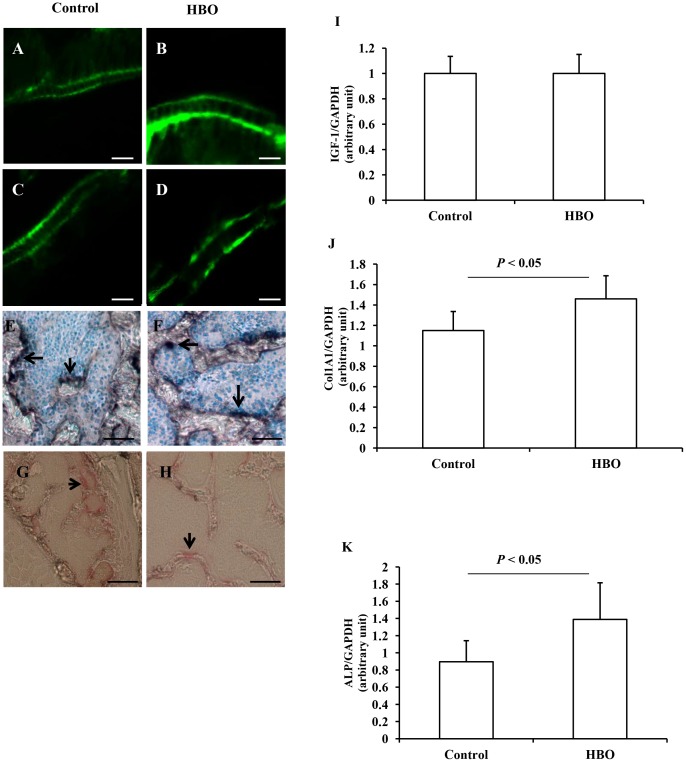
Detection of calcein-labeled mineralized fronts, ALP- and TRAP-positive cells, and changes in the expression of IGF-1, Col1A1, and ALP mRNA. Two calcein-labeled mineralized fronts are visualized by fluorescent micrography in the femur (A) and the first lumbar vertebra (C) of the control group, and the femur (B) and the first lumbar vertebra (D) of the HBO group. ALP-positive osteoblasts in the first lumbar vertebra of the control (E) and HBO (F) groups are stained black (arrows) and nuclei are stained blue. TRAP-positive osteoclasts in the first lumbar vertebra of the control (G) and HBO (H) groups are stained red (arrows). Scale bars for A-D, 10 µm; for E-H, 100 µm. Exposure to HBO did not increase the expression of IGF-1 mRNA levels (I), but increased the expression of Col1A1 (J) and ALP (K) mRNA levels.

The MAR in the femur and first lumbar vertebra was significantly higher in the HBO group than in the control group (Figures 3A-D; Table 1). Similarly, the BRF/BS in the femur and first lumbar vertebra were significantly higher in the HBO group than in the control group (Table 1). Body weight showed similar changes in both groups during the experimental period (Table 1). Daily food consumption was also similar (data not shown). The Ob.S/BS and Oc.S/BS in the first lumbar vertebra were approximately similar in the two groups (Figures 3E-H; Table 1).

**Table 1:** Effects of HBO on body weight, MAR, BFR/BS, Ob.S/BS, and Oc.S/BS.

	control group	HBO group
body weight (g)		
Pre-experiment	19.6±1.1	20.7±1.4
Post-experiment	21.4±1.0#	22.1±1.0#
femur MAR (µm/day)	1.04±0.12	1.38±0.26**
lumbar vertebra MAR (µm/day)	1.55±0.20	2.04±0.21**
femur BFR/BS (mm^3^/mm^2^/year)	0.110±0.024	0.185±0.065**
lumbar BFR/BS (mm^3^/mm^2^/year)	0.154±0.058	0.254±0.103*
Ob. S/BS (%)	16.1±3.0	15.6±3.7
Oc. S/BS (%)	4.8±0.9	4.4±1.3

Values are expressed as means ± SD. #Significant differences (*P*<0.01) compared with pre-experiment levels. **Significant differences (*P*<0.01) compared with the control group. *Significant differences (*P*<0.05) compared with the control group.MAR, mineral apposition rate; BFR/BS, bone formation rate/bone surface; Ob.S/BS, percentage of bone surface covered by osteoblasts; Oc.S/BS, percentage of bone surface covered by osteoclasts. n = 8 for each group.

### Effects of HBO on the expression of IGF-1, Col1A1, and ALP mRNA

The IGF-1 mRNA level was similar in the control and HBO groups (Figure 3I). However, the Col1A1 and ALP mRNA levels were significantly increases in the HBO group compared with the control group (Figures 3J, K).

### Effects of HBO on callus formation and fracture healing

**Figure 4:**
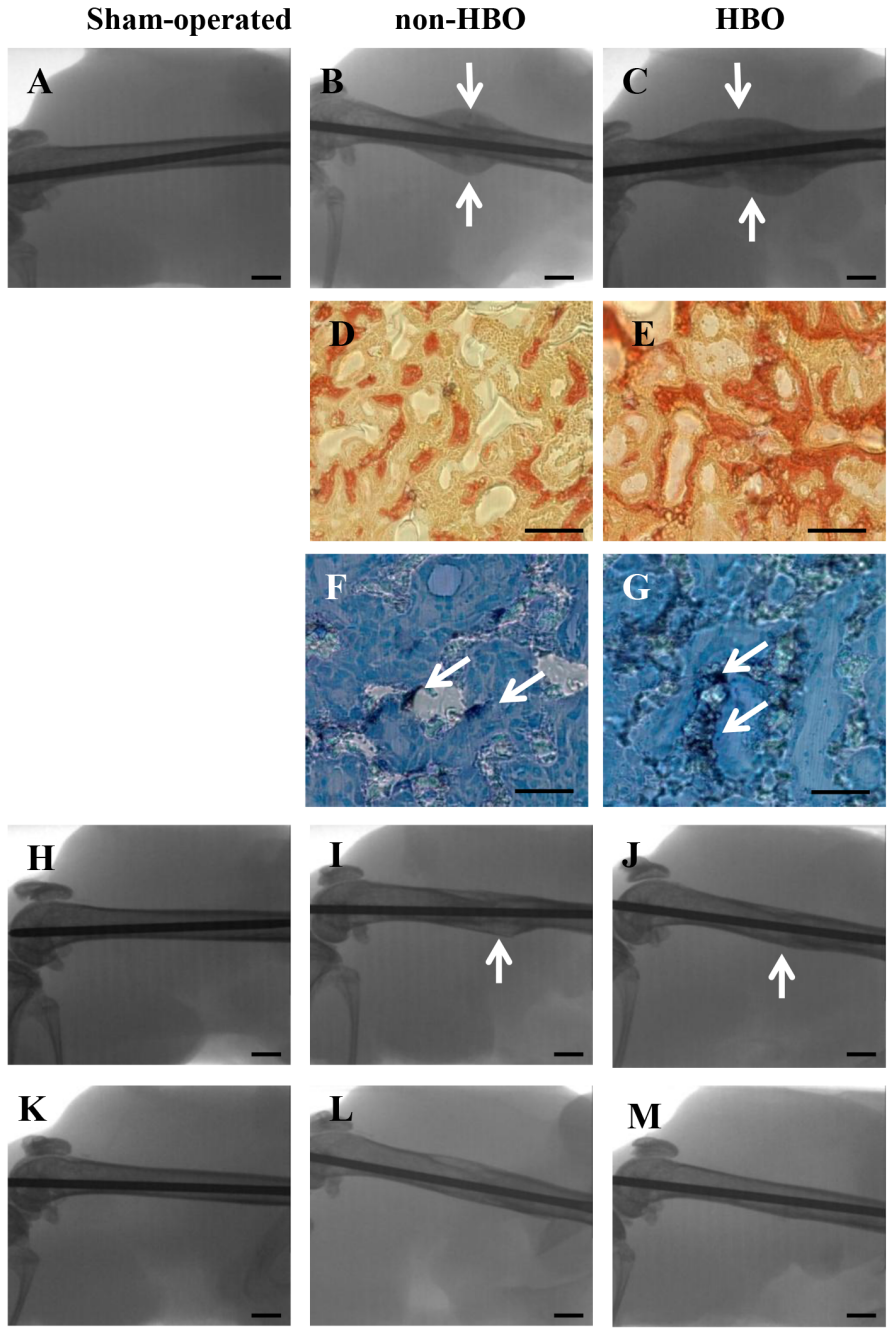
Effects of HBO on fracture healing. Radiographs of the healing femurs at 2 (A-C), 4 (H-J), and 6 (K-M) weeks after fracture. Arrows indicate the callus (B, C, I, J). Collagen fibers of the osteoid in the callus were stained red by Van Gieson’s staining at 2 weeks after fracture (D, E). ALP-positive osteoblasts in the callus of the control (F) and HBO (G) groups are stained black (arrows) and nuclei are stained blue. Histomorphometric analysis of callus size was performed in relation to the diameter of the femur at the fracture site at 2 weeks after fracture (B, L, n = 8 for each group). Scale bars for radiographs, 1 mm; for Van Gieson’s and ALP staining, 100 µm.

Fracture healing was visually assessed using radiographs. At 2 weeks after fracture, no radiographic differences in fracture healing were observed between the non-HBO and HBO groups, but the callus area was significantly larger in the HBO group than in the non-HBO group (Figures 4B, C; Table 2). Van Gieson’s staining revealed osteoid formation in the callus (Figures 4D, E). The percentage area of osteoid/callus was significantly greater in the HBO group than in the non-HBO group (Figures 4D, E; Table 2). Similarly, Ob.S/BS in the callus was significantly higher in the HBO group than in the control group (Figures 4F, G; Table 2). At 4 weeks after fracture, the callus in the HBO group had almost disappeared, whereas a small area remained in the non-HBO group (Figures 4I, J). At 6 weeks after fracture, cortical bone at the fracture site in the non-HBO group was rather obscure, whereas accelerated fracture healing was radiographically observed in the HBO group compared with the non-HBO group (Figures 4L, M). No orthopedic changes were observed at any point in the sham-operated group (Figures 4A, H, K). At 2 and 4 weeks after fracture, the increase in body weight was similar in all groups (Table 2). However, at 6 weeks, the body weight was significantly lower in the non-HBO and HBO groups than in the sham-operated group (Table 2). The body weight at 6 weeks showed a condition × time interaction. Food consumption was similar in all groups throughout the experimental period (data not shown).

**Table 2:** Changes in body weight and callus, and concentrations of urinary 8-OHdG, DPD and plasma calcium.

	Sham-operated	non-HBO	HBO
body weight (g)			
Pre-experiment	23.0±1.0	22.8±0.9	22.5±1.1
2-wk postfracture	24.5±0.9*	24.9±1.0*	23.8±1.6*
Pre-experiment	24.9±0.9	22.5±0.6	24.2±0.7
4-wk postfracture	28.3±0.9*	26.9±0.7*	27.4±1.0*
Pre-experiment	24.1±0.7	23.8±1.2	24.1±1.3
6-wk postfracture	29.5±1.1*	27.3±0.9*†	27.1±1.3*†
callus area/femur diameter (mm)	—	2.61±1.09	4.12±1.18♦
osteoid area/callus (%)	—	18.3±3.6	27.3±8.8♦
callus Ob.S/BS (%)	—	13.0±2.8	19.8±2.8♦
urinary 8-OHdG (ng/mL)	19.3±15.1	14.0±10.6	10.5±7.3
urinary DPD (nmol/L)			
2-wk postfracture	86.2±18.0	136.2±70.8	157.6±77.0†
4-wk postfracture	68.0±25.1	76.1±10.5#	114.9±95.7
6-wk postfracture	68.2±30.4	74.5±30.5#	78.1±23.9#
plasma calcium (mg/dL)			
2-wk postfracture	9.52±0.33	10.02±0.66	9.33±0.74
4-wk postfracture	9.44±0.71	9.65±0.97	9.38±0.53
6-wk postfracture	9.53±1.06	10.40±0.85	9.49±0.98

Values are expressed as means ± SD. *Significant differences (*P*<0.05) compared with pre-experiment levels. †Significant differences (*P*<0.05) compared with the sham-operated group. ♦Significant differences (*P*<0.05) compared with the non-HBO group. # Significant differences (*P*<0.05) compared with levels at 2 weeks after fracture. n = 8, n = 4, and n = 12 for each group at 2, 4, and 6 weeks after fracture.

### Biomechanical testing

**Figure 5:**
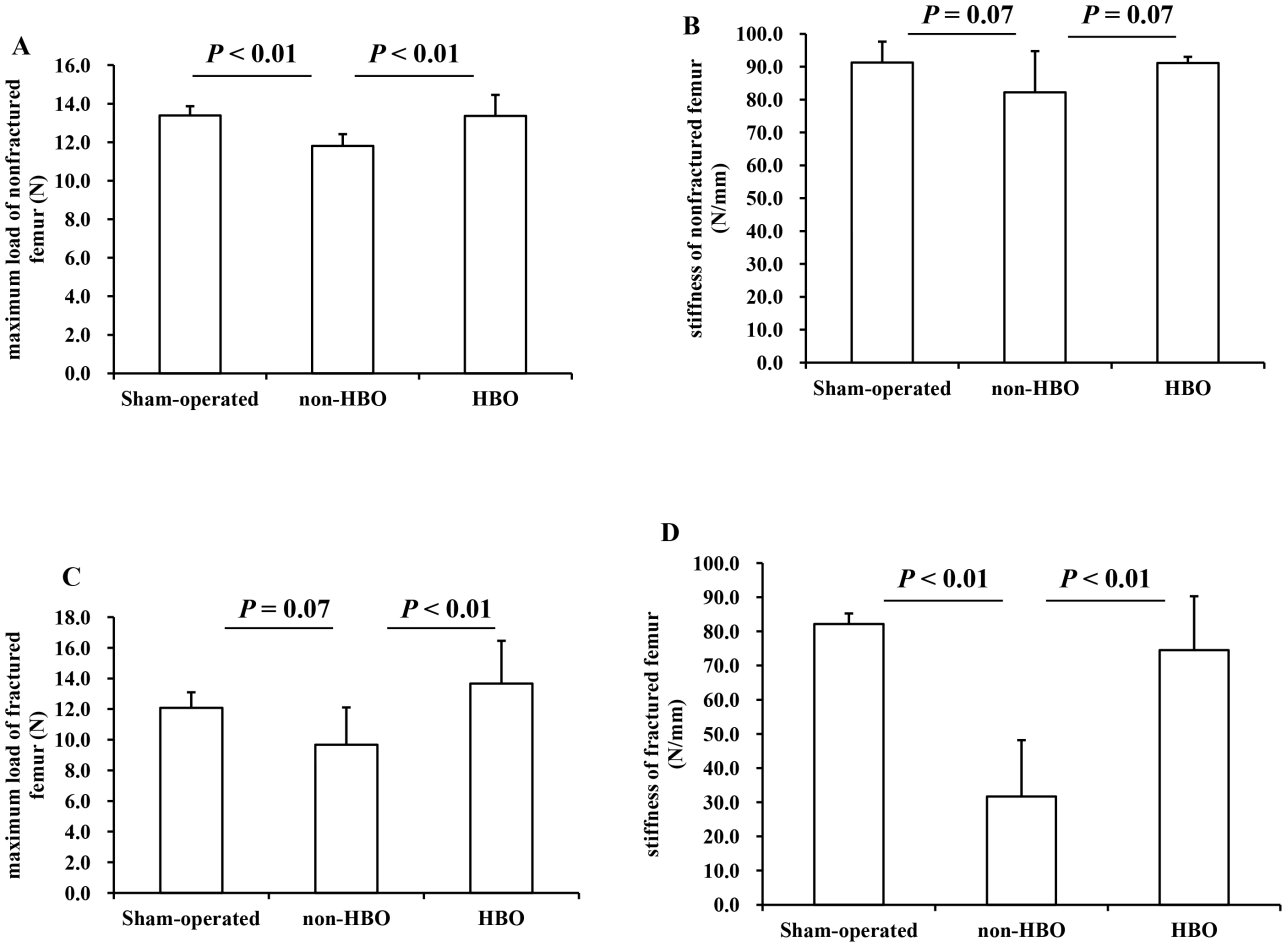
The maximum load and stiffness values for the nonfractured left femur (A, C) and the fractured right femur (C, D) at 6 weeks after fracture are shown (n = 6 for each group).

At 6 weeks after fracture, the maximum load borne by the nonfractured (contralateral) femur was significantly lower in the non-HBO group than in the sham-operated and HBO groups (Figure 5A). The stiffness of the contralateral femur tended to be lower in the non-HBO group than in the sham-operated and HBO groups, but the difference was not significant (*P* = 0.07; Figure 5B). The maximum load borne by the fractured femur in the non-HBO group was significantly lower than that in the HBO group (Figure 5C) and insignificantly lower than that in the sham-operated group (*P* = 0.07; Figure 5C). Further, the stiffness of the fractured femur was significantly lower in the non-HBO group than in the sham-operated and HBO groups (Figure 5D).

### Urine and blood analyses

Urinary 8-OHdG level at 6 weeks after fracture did not show significant differences between the groups (Table 2). At 2 weeks after fracture, the DPD level was significantly higher in the HBO group than in the sham-operated group (Table 2). In the non-HBO group, the DPD level was significantly lower at 4 weeks than at 2 weeks after fracture. In the non-HBO and HBO groups, the DPD level was significantly lower at 6 weeks than at 2 weeks after fracture (Table 2). In the sham-operated group, the DPD level was relatively constant (Table 2). The plasma calcium concentrations were constant in all groups (Table 2).

## Discussion

Bone formation is controlled by several factors, of which osteoblast proliferation, type 1 collagen synthesis, and osteoid calcification are important. IGF-1 stimulates osteoblast proliferation in vitro and in vivo [11], [17]–[19]. Osteoblasts synthesize and secrete type 1 collagen, a main component of osteoid, and osteoid is calcified by ALP, which leads to bone formation [20]. In the present study, we used HBO treatments to influence bone formation. Exposure to hyperoxia and/or hyperbaric conditions increases the dissolved oxygen content in vivo [21], [22]. Although HBO treatments did not increase IGF-1 mRNA expression and Ob.S/BS, MAR and BRF increased significantly. When osteoblasts proliferate proportionally with bone growth, Ob.S/BS is considered to be constant. Therefore, the HBO conditions in this study may have influenced osteoblast proliferation to some extent. In addition, at 2 weeks after fracture, Ob.S/BS in the callus increased significantly in the HBO group compared with that in the non-HBO group. These results imply that HBO conditions stimulate osteoblast proliferation in vivo.

ALP activity has been shown to be upregulated by exposure to HBO in vitro in different cell lines and under different HBO conditions [4], [5]. Wu et al. reported that HBO upregulated ALP activity and calcium deposition in vitro [5]. By contrast, hypoxia sharply reduces osteoblast ALP activity and ALP mRNA expression in vitro [6]. These findings indicate that the expression and activity of ALP are sensitive to oxygen tension. Our in vivo study showed that ALP mRNA expression increased after exposure to HBO. The extracellular matrix of bone consists of almost 90% type 1 collagen [20], and hyperoxia increases collagen synthesis in vitro [4]. In this study, exposure to HBO increased in vivo Col1A1 mRNA expression, MAR and BRF. Our results and those of other studies show that the appropriate HBO conditions can partially enhance bone anabolism in vivo.

Bone anabolism is important for rapid fracture healing. Because the present HBO conditions were confirmed to enhance bone anabolism in vivo, we assessed the effect of these conditions on fracture healing. In experimental animal models, the callus volume around the fracture site peaks at approximately 2 weeks after fracture [1], [2]. Moreover, poor callus formation delays fracture healing [2]. Lue et al. reported a unique animal model of ischemic fracture in which the femoral artery is resected to create hypoxic conditions at the fracture site [23]. The ischemic fracture impairs callus formation and delays fracture healing, but under hyperoxic condtions in a chamber with 50% oxygen, the impairment is inhibited and fracture healing is accelerated [24]. In this study, the radiographic findings did not show any obvious differences in fracture healing between the HBO and non-HBO groups at 2 weeks after fracture; however, callus formation was significantly greater in the HBO group than in the non-HBO group. Enhanced osteoid formation and increased Ob.S/BS were observed in the callus only in the HBO group. Furthermore, urinary DPD, a biomarker of bone resorption, was higher in the HBO group than in the sham-operated group 2 weeks after fracture. Our results and those of other studies imply that HBO treatment enhances bone anabolism and turnover in the early phase of fracture healing.

At 4 and 6 weeks after fracture, the radiographic findings showed accelerated fracture healing in the HBO group compared with the non-HBO group. To clarify these findings in detail, biomechanical measurements were performed on femurs from each group at 6 weeks after fracture. Although the left femur was intact, the maximum load borne by the left femur was significantly lower in the non-HBO group than in the sham-operated and HBO groups, implying that unilateral femur fracture impairs whole-body bone biomechanical growth. Similarly, bone stiffness tended to be lower in the non-HBO group than in the sham-operated and HBO groups. Although food consumption was similar in all groups throughout the experimental period (data not shown), body weight was significantly lower in the non-HBO and HBO groups than in the sham-operated group at 6 weeks after fracture, implying that mice were exposed to severe stress due to the fracture. Regardless of the impaired increase in body weight in the HBO group, the biomechanical strength of the nonfractured femur was not impaired compared with the sham-operated group, implying that exposure to HBO and the resultant bone anabolism normally regulated the biomechanical growth of the nonfractured femur. The maximum load borne by the fractured femurs was significantly lower in the non-HBO group than that in the HBO group. Similarly, the stiffness of the fractured femurs was significantly lower in the non-HBO group than in the sham-operated and HBO groups. These results indicate that the HBO treatments in this study accelerated fracture healing. However, further studies are required to clarify the molecular mechanisms underlying fracture healing under HBO conditions before this treatment can be applied in humans.

In this study, plasma calcium concentrations were constant in all groups throughout the study, indicating that HBO treatment accelerated fracture healing without disruption of calcium homeostasis. Moreover, the urinary 8-OHdG level did not differ between the groups at 6 weeks after fracture. This indicates that abnormal DNA oxidative damage was not induced by the present HBO treatments.

In conclusion, this study indicates that HBO treatment increases MAR and BRF in vivo. Furthermore, we demonstrated that HBO treatment histologically and biomechanically accelerates fracture healing in vivo without disrupting calcium homeostasis and causing abnormal DNA oxidative damage. We found that a unilateral fracture impaired the biomechanical strength of the contralateral bone and that HBO exposure corrected this impairment. These results indicate that HBO treatment is beneficial for fracture healing. However, whether increased oxygen tension directly influences bone anabolism remains unclear. The details of this molecular mechanism remain to be established.
